# Social media–related dietary behaviors and integrated behavioral model constructs among university students: A cross-sectional study

**DOI:** 10.1371/journal.pone.0362745

**Published:** 2026-11-12

**Authors:** Amani Alhazmi, Maha Ali, Manal Mohammed Hawash, Bali Sharma, Ali Mohieldin, Mahmoud Abdelwahab Khedr

**Affiliations:** 1 Department of Public Health, Applied Medical Sciences College, King Khalid University, Abha, Saudi Arabia; 2 Department of Basic Medical Sciences, College of Applied Medical Sciences, King Khalid University, Abha, Saudi Arabia; 3 College of Nursing, University of Hafr Al Batin, Hafar Al Batin, Eastern Province, Saudi Arabia; Healthcare and Technology Institute (HCT Academy), PHILIPPINES

## Abstract

**Background:**

Social media exposes young adults to nutrition information, food marketing, peer norms, and health-related content. However, evidence on social media–related dietary behaviors and their links to Integrated Behavioral Model (IBM) constructs among Saudi university students is limited. This study examined these behaviors, IBM constructs, and factors associated with the Dietary Habits score among King Khalid University (KKU) students.

**Methods:**

A cross-sectional study included 385 KKU undergraduates, recruited through convenience sampling between February and April 2026. Participants completed the Arabic IBM-based Social Media and Dietary Habits Questionnaire. Analyses included descriptive statistics, Spearman’s correlation, independent-samples t-tests, one-way ANOVA with Tukey’s post hoc tests, and multiple linear regression. Regression coefficient p-values were adjusted using the Holm–Bonferroni procedure across the 32 coefficient tests.

**Results:**

The mean dietary habits score was 12.06 ± 4.17. All IBM constructs were positively correlated with dietary habits (p < 0.001). Exploratory comparisons showed that Dietary Habits scores differed by gender, marital status, BMI, chronic illness, physical activity, smoking, sleep duration, healthy diet, and social media use. The regression model explained 57.6% of the variance in dietary scores (adjusted R² = 0.537). After Holm adjustment, higher scores were linked to attitude (B = 0.199, 95% CI: 0.079–0.319; adjusted p = 0.034), physical activity (B = 1.295, 95% CI: 0.536–2.054; adjusted p = 0.026), and social media use of 1–2 hours/day (B = 2.005, 95% CI: 0.921–3.089; adjusted p = 0.010) or 3–4 hours/day (B = 1.220, 95% CI: 0.511–1.929; adjusted p = 0.024), compared with >4 hours/day.

**Conclusions:**

IBM constructs were positively interrelated and associated with social media–related dietary behaviors. Positive attitudes, physical activity, and moderate daily social media use were associated with higher Dietary Habits scores. These findings suggest that behavioral beliefs and lifestyle context may be relevant in the design of digital nutrition programs for university students.

## Introduction

The transition to university represents a significant shift in young people’s eating behaviors. Away from the family home, many students prepare and eat their own meals for the first time, and their dietary decisions often become entrenched and persist after graduation. The habits young people form during these formative years may manifest as weight gain in adulthood and subsequently result in noncommunicable diseases (NCDs), such as type 2 diabetes, cardiovascular disease, and various types of cancer [1,2]. The World Health Organization reported that more than one billion people were living with obesity in 2022 and that worldwide adult obesity had more than doubled since 1990 [3]. Understanding what is likely to influence young adults’ eating habits toward or away from healthy eating during university years is not a narrow academic question but a matter of population health. This is most evident in the Gulf, and especially in Saudi Arabia. The Kingdom has experienced an increase in the number of obese people in a single generation, due to rapid urbanization, increased household income, and a resulting shift toward diets high in refined carbohydrates, sugar, and saturated fat [4]. Obesity affects roughly a quarter to a third of adults in the Kingdom, with being overweight affecting a comparable share again, and the burden of NCDs is emerging as one of the main public-health challenges that the country will be faced with through the reforms outlined in Vision 2030 [4,5]. Diet is central to this burden, making young people’s eating behaviors an important focus for researchers and policymakers [3].

Surveys of students in Saudi Arabia raise significant concerns. Combined overweight and obesity occur in approximately one-third or more of students in cross-sectional studies at both university and medical levels, where breakfast skipping is common, and intake of fruit and vegetables is low [5–7]. Limited knowledge alone is unlikely to explain these patterns. They reflect the food context in which students eat, the time demands and economic constraints of student life, the breakdown of social structure around mealtimes when students live away from home, and the interplay of social and psychological factors that shape what students eat [6,7]. Social media has become an increasingly relevant influence within this context. Saudi Arabia has one of the most connected populations in the world. Internet penetration is close to saturation, with the number of social media accounts over 30 million, and adults spending on the order of 2.5 hours a day on social platforms plus much more time online in general [8]. The younger generation of Saudis drives this activity and can seamlessly navigate platforms such as WhatsApp, Instagram, Snapchat, TikTok, and YouTube, with Snapchat and Instagram use far higher than the global average [9]. Food is woven at every turn into the feed that students follow: recipe reels and cooking videos, restaurant reviews, calorie-counted ‘what I eat in a day’ posts, influencer promotions, and a steady stream of snack, delivery, and soft-drink ads. Young people are no longer exposed to food-related content only when it’s convenient; it has become an ambient part of everyday life.

Several pathways may link exposure to food-related social media content with food norms and choices, including visual cues, social comparison, peer endorsement, influencer promotion, and direct advertising of energy-dense products [10,11]. Saudi evidence points in this direction. Aljefree and Alhothali (2022) reported that university students in Jeddah who more frequently purchased food after viewing social media advertisements also consumed more potato chips and fast food and had a higher prevalence of obesity. Research on Saudi social media influencers has also identified associations between followers’ food choices and influencer content [12], and a separate national study reported a greater preference for nutrient-poor products among respondents engaging with food-related online content [13]. Findings from college populations elsewhere similarly associate exposure to food marketing with body mass index [14].

The relationship is not uniformly adverse. Social media platforms also share nutrition information, healthy recipes, fitness content, and peer support, and researchers have used them to deliver healthy-eating interventions to young adults [1,13]. At the same time, image-centered platforms have been associated with body dissatisfaction, upward social comparison, and disordered or emotional eating, particularly among young women [11,15]. These contrasting findings suggest that food-related content, individual interpretation, and the surrounding social context may be as important as time spent online. Accordingly, the present study focuses on self-reported dietary behaviors specifically related to social media and the behavioral constructs that accompany them, rather than treating screen time as a direct measure of dietary quality.

A theoretical framework can help organize these related behavioral factors. The Theory of Reasoned Action and the Theory of Planned Behavior propose that behavior is proximally related to intention, which is shaped by attitudes, perceived norms, and perceived behavioral control [16,17]. The Integrated Behavioral Model (IBM) combines constructs from these and other behavioral theories. Within the IBM, intention is shaped by attitude, perceived norms, and personal agency, while knowledge and skills, salience, environmental constraints, and habit may affect whether an intended behavior is performed [17,18]. In a cross-sectional study, these constructs can be examined as theoretically informed correlates, but their temporal or causal ordering cannot be established.

Studies based on reasoned action models have shown that attitudes, self-efficacy, perceived control, and intention are associated with dietary intentions and behaviors [19,20]. In the context of social media, these constructs provide a theoretical framework for examining how students evaluate nutrition-related content, perceive others’ expectations and practices, assess their ability to act on dietary information, and report related intentions, knowledge, skills, and behaviors. The present study therefore examines the pattern of associations among these constructs without attempting to establish causal or mediating pathways.

Food practices in Saudi Arabia do not map neatly onto the settings where most of this research was carried out. Eating is tied to family and communal eating, to hospitality, and to gendered patterns of social life, and the platforms people spend most time on locally, Snapchat and Instagram, among them, are strongly visual [9]. How descriptive and injunctive norms, body-image pressures, and influencer content play out under these conditions need not resemble what has been reported for North American and European samples, and that is a large part of why locally grounded, theory-based evidence must be generated rather than assumed from elsewhere.

Theory-informed evidence linking social media with dietary behavior remains limited in the Gulf region. Most Saudi studies have described associations between social media exposure, food marketing, and dietary choices without jointly examining a broader set of behavioral constructs [9,12,13]. An IBM-based Social Media and Dietary Habits Questionnaire was recently developed and validated among Saudi university students [21]. Applying this instrument in a different university population may provide additional evidence regarding the distribution, reliability, and correlates of its construct scores.

The present study examined social media–related dietary behaviors and IBM constructs among undergraduate students at King Khalid University (KKU), Saudi Arabia. Specifically, the study aimed to: (i) describe participants’ social media use, social media–related Dietary Habits scores, and IBM construct scores; (ii) examine the relationships between the Dietary Habits score and the IBM constructs; and (iii) identify sociodemographic, health, lifestyle, social media use, and IBM factors associated with the Dietary Habits score. The findings are intended to provide context-specific, theory-informed evidence that may guide future longitudinal research and the development of culturally appropriate digital nutrition communication strategies delivered through social media as a digital health platform.

## Methodology

### Study design and participants

An analytical cross-sectional study was conducted to examine social media–related dietary behaviors and their associations with IBM constructs among undergraduate students at KKU in Abha, Saudi Arabia. Data were collected from February 9 to April 11, 2026, using an anonymous online survey that included a validated questionnaire developed within the IBM framework [18,21]. King Khalid University is a large public university in the southern region of Saudi Arabia and includes colleges across health sciences, natural sciences, engineering, humanities, social sciences, and administrative disciplines. The study population consisted of male and female undergraduate students enrolled at KKU during the study period, regardless of academic specialization. Students were eligible if they were officially enrolled undergraduate students aged 18 years or older, able to complete the Arabic questionnaire, and willing to provide electronic informed consent.

A non-probability convenience sampling technique was applied [22]. Participants were recruited by electronically distributing the survey link via social media platforms, including WhatsApp, Telegram, and X (formerly Twitter), university mailing lists, and departmental communication groups across different colleges of KKU. The survey link was also shared through student representatives and class leaders to maximize participation across academic disciplines and study levels.

According to KKU’s official academic statistics, the total undergraduate student population during the study period was approximately 61,708 students. The minimum required sample size was calculated using the Raosoft Sample Size Calculator based on a 95% confidence level, a 5% margin of error, and a response distribution of 50%, resulting in a minimum required sample size of 382 participants [23]. The recruitment target was increased by approximately 10% to 420 participants to allow for potential non-response and incomplete questionnaires. The final analytical sample comprised 385 eligible students with complete responses, exceeding the calculated minimum of 382. The 40 pilot participants were excluded from the analytical sample. The survey link was distributed through multiple open communication channels; therefore, the total number of students who received the invitation could not be determined. The numbers of those who accessed the survey but declined participation, or were excluded before inclusion, were not recorded; consequently, the complete recruitment flow and the overall response rate could not be verified.

The original sample-size calculation addressed precision for estimating a proportion and did not establish power for multiple regression. During revision, an analysis-specific sensitivity assessment was performed using the sample size achieved of 385 participants. The calculation used the noncentral F distribution for the overall test of R² = 0 in an ordinary least-squares linear model with 32 slope parameters plus an intercept (numerator degrees of freedom = 32; residual degrees of freedom = 352), a significance level of α = 0.05, and desired power of 80%. Assumptions were a full-rank fixed predictor matrix and independent, normally distributed errors with constant variance. Cohen’s f² was defined as R²/(1 − R²), with noncentrality parameter λ = Nf². Solving for 80% power gave a minimum detectable overall effect of f² = 0.0697 (approximately 0.070), equivalent to R² = 0.0652. This was a sensitivity assessment based on the achieved sample size, not an a priori sample-size calculation or a calculation using the observed model effect. It concerns the overall model and does not establish adequate power or precision for individual coefficients, particularly for uncommon categories or correlated IBM constructs.

### Study instrument

Data were collected using a structured, self-administered questionnaire consisting of two sections. The questionnaire was administered electronically using Google Forms and was designed to collect information on participants’ sociodemographic characteristics, health and lifestyle factors, social media use, and social media–related dietary behaviors and the IBM. The Arabic version of the questionnaire was used because Arabic is the native language of the study population. The original Arabic version, developed and psychometrically validated by Bawazeer (2025), was adopted; it was specifically designed to assess social media–related dietary behaviors and associated IBM among university students using the IBM framework [21].

The first section collected participants’ sociodemographic and health-related information, including age (18–22, 23–25, or >25 years); gender (female or male); marital status (unmarried or married); living arrangement (with family, alone, or with friends); monthly family income sufficiency (sufficient or insufficient); college type (health, science and engineering, humanities and social sciences, or community and applied colleges); academic level (first, second, third, fourth, or internship year); diagnosed chronic disease (yes or no); physical activity (yes or no); smoking status (smoker or non-smoker); sleep duration (≤6, 7–8, or >8 hours per night); and following a specific healthy diet (yes or no). Additional information regarding the participants’ social media usage patterns, including the average number of hours spent daily on social media platforms (<1, 1–2, 3–4, or >4 hours), was collected because prolonged exposure to social media has been reported to be associated with dietary choices and health behaviors among young adults. Body mass index was calculated as weight in kilograms divided by height in meters squared (kg/m²) and classified according to the World Health Organization (WHO) criteria into underweight (<18.5 kg/m²), normal weight (18.5–24.9 kg/m²), overweight (25.0–29.9 kg/m²), and obesity (≥30.0 kg/m²) [24].

The second section consisted of the original Arabic version of the Social Media and Dietary Habits Questionnaire, developed according to the IBM to assess social media–related dietary behaviors and IBM constructs [21]. The questionnaire contains 38 items distributed across eight IBM constructs: Dietary Habits (5 items), Attitude (5 items), Perceived Norms (6 items), Personal Agency (5 items), Intention (5 items), Salience (4 items), Skills (4 items), and Knowledge (4 items). Collectively, these constructs assess the behavioral, cognitive, motivational, social, and environmental determinants through which social media may influence healthy dietary behaviors among university students. The first construct, Dietary habits, assessed the frequency of healthy dietary behaviors that may be influenced by social media, including meal regularity, adherence to healthy dietary patterns promoted through social media, healthy dining choices, meeting daily nutritional recommendations, and trying healthy recipes obtained from social media platforms. Participants responded using a five-point Likert scale ranging from 1 (Never) to 5 (Always). The second construct, Attitude, evaluated participants’ attitudes toward the influence of social media on healthy dietary behaviors using five statements rated on a five-point Likert scale ranging from 1 (Strongly disagree) to 5 (Strongly agree). The third construct, Perceived Norms, consisted of six items assessing perceived social influences related to dietary behaviors through social media. Responses were recorded on a five-point Likert scale ranging from 1 (Strongly disagree) to 5 (Strongly agree). The fourth construct, Personal Agency, measured participants’ confidence in their ability to perform healthy dietary behaviors with the support of social media. These five items used confidence responses, coded from 1 (Not confident) to 5 (Strongly confident). The fifth construct, Intention, evaluated participants’ intentions to use social media to adopt healthier dietary behaviors. Participants rated five items on a five-point Likert scale ranging from 1 (Strongly disagree) to 5 (Strongly agree). The sixth construct, Salience, assessed the perceived importance of maintaining healthy dietary behaviors influenced by social media. Four items were measured using a five-point Likert scale ranging from 1 (Strongly disagree) to 5 (Strongly agree). The seventh construct, Skills, evaluated participants’ self-perceived nutrition-related skills acquired through social media. Four items were scored using a binary response format (Yes = 1; No/Not sure = 0). The eighth construct, Knowledge, assessed participants’ nutritional knowledge acquired through social media using four binary-response items (Yes = 1; No/Not sure = 0).

Each domain score was obtained by summing its corresponding responses. Possible ranges were 5–25 for Dietary Habits, Attitude, Personal Agency, and Intention; 6–30 for Perceived Norms; 4–20 for Salience; and 0–4 for Skills and Knowledge. Higher scores reflected more favorable self-reported social media–related dietary behaviors and IBM construct scores. Scores were analyzed as continuous variables to preserve statistical power because established categorical thresholds were unavailable [25]. The original questionnaire demonstrated excellent psychometric properties. Content validity was established through expert review, whereas construct validity was confirmed using exploratory factor analysis with principal component extraction and Varimax rotation. The Kaiser–Meyer–Olkin (KMO) measure of sampling adequacy was 0.937, and Bartlett’s test of sphericity was statistically significant (p < 0.001), confirming the data’s suitability for factor analysis. The eight extracted factors explained 64.5% of the total variance, supporting the theoretical structure of the Integrated Behavioral Model. Factor loadings indicated that all questionnaire items loaded satisfactorily onto their corresponding theoretical constructs. The original instrument also demonstrated excellent internal consistency reliability, with an overall Cronbach’s alpha coefficient of 0.949, whereas domain-specific Cronbach’s alpha coefficients ranged from 0.701 to 0.935, indicating acceptable to excellent reliability across all questionnaire domains [21].

The questionnaire was pilot tested among 40 undergraduate students who were not included in the final analytical sample. The pilot assessed clarity, readability, comprehension, electronic functionality, and completion time, which averaged 10–15 minutes. Following pilot testing, the questionnaire items, response options, scoring procedures, and conceptual meanings remained unchanged; no items were added or deleted. Consistent with standard methodological recommendations for pilot studies, data from pilot participants were excluded from the final statistical analysis [26]. Domain-specific Cronbach’s alpha coefficients in the analytical sample were 0.815 for Dietary Habits, 0.924 for Attitude, 0.903 for Perceived Norms, 0.915 for Personal Agency, 0.931 for Intention, 0.876 for Salience, 0.817 for Skills, and 0.783 for Knowledge, indicating acceptable to excellent internal consistency of the separate domain scores in this sample.

### Ethical considerations

Ethical approval for the study was obtained from the Institutional Review Board of King Khalid University, Abha (Approval No. KKU-35-2026-6). Participation was voluntary, and all participants provided electronic informed consent before completing the questionnaire. Participants indicated consent by selecting the “Agree” option before beginning the survey. Participants could choose whether to complete the survey, and incomplete responses were excluded from the analysis. The study maintained participants’ anonymity and confidentiality, and no personally identifiable information was collected. All collected data were used solely for research purposes and stored securely. All study procedures were conducted in accordance with the ethical principles of the Declaration of Helsinki.

### Statistical analysis

Statistical analysis was performed using IBM SPSS Statistics for Windows, Version 27.0 (IBM Corp., Armonk, NY, USA) [27]. Prior to analysis, the dataset was screened for completeness, duplicate responses, coding errors, missing values, outliers, and logical inconsistencies. Only questionnaires with complete responses fulfilling all eligibility criteria were included. Continuous variables were examined for normality using visual inspection of histograms, Q–Q plots, and the Shapiro–Wilk test, whereas homogeneity of variances was assessed using Levene’s test [28,29]. Descriptive statistics were used to summarize participants’ characteristics. Categorical variables were presented as frequencies and percentages, whereas continuous variables were summarized using means and standard deviations (SD), medians, ranges, and 95% normal-approximation confidence intervals for means. Domain-specific internal consistency was evaluated using Cronbach’s alpha coefficients [30]. The relationships among IBM constructs were examined using Spearman’s rank correlation coefficient (ρ), with the absolute magnitude of coefficients interpreted according to Cohen’s criteria (0.10–0.29 weak, 0.30–0.49 moderate, ≥ 0.50 strong) [25]. Spearman’s correlation was selected to accommodate the ordinal response formats underlying the domain scores and potential departures from normality. Differences in social media-related Dietary Habits scores according to participants’ characteristics were examined using Welch’s independent-samples t-tests for dichotomous variables and one-way ANOVA for variables with more than two categories. For statistically significant ANOVA results, Tukey’s honestly significant difference (HSD) post hoc test was used to identify specific pairwise group differences [31]. Effect sizes were reported as Cohen’s d for t-tests and eta squared (η²) for ANOVA, interpreted according to Cohen’s criteria [25].

The primary analysis used multiple linear regression with ordinary least squares, with the Dietary Habits score as the dependent variable and all predictors entered simultaneously using the Enter method. The model included all recorded participant characteristics: age group, gender, marital status, living arrangement, family income sufficiency, BMI category, college type, academic level, diagnosed chronic disease, physical activity, smoking, daily social media use, sleep duration, together with the seven remaining IBM constructs (Attitude, Perceived Norms, Personal Agency, Intention, Salience, Skills, and Knowledge). Following a healthy diet was excluded because of its conceptual overlap with the outcome. No stepwise selection or screening based on univariable p-values was applied. Categorical predictors were represented by dummy variables, yielding 32 slope parameters plus an intercept. The reference categories were age 18–22 years, female gender, unmarried status, living with family, sufficient family income, normal BMI (18.5–24.9 kg/m²), health colleges, fourth academic year, no diagnosed chronic disease, no physical activity, non-smoker status, social media use > 4 hours/day, and sleep duration ≤6 hours/night.

Multicollinearity was assessed using variance inflation factors (VIFs) and tolerance values. VIF ≥ 5 or tolerance ≤0.20 was used as a conservative threshold warranting caution regarding multicollinearity rather than relying on VIF < 10. These thresholds were treated as diagnostic flags and did not establish coefficient stability. Predictor-specific VIF and tolerance values are reported in Table 5. Regression assumptions were assessed using residual plots and standardized residuals, with no major violations identified. Model fit was evaluated using R², adjusted R², and the overall F-test. Results included unstandardized coefficients (B), standardized coefficients (β), standard errors, 95% confidence intervals, t-statistics, and p-values.

Holm–Bonferroni adjustment was applied across the 32 slope-coefficient tests. Both nominal and adjusted p-values were reported, and statistical significance of individual regression coefficients was assessed using Holm-adjusted p-values <0.05. The adjustment was applied to p-values; regression confidence intervals were conventional 95% intervals unadjusted for multiplicity. Correlations and overall group comparisons were treated as exploratory analyses. Tukey’s HSD adjusted pairwise p-values and 95% confidence intervals within each factor but did not control multiplicity across different factors or analyses. These procedures addressed inflated Type I errors within the specified test families rather than across all analyses in the study. All tests were two-tailed; nominal p-values <0.05 were used for exploratory correlations and overall group comparisons. The designation of regression as the primary analysis and correlations and group comparisons as exploratory analyses was determined during revision and was not prospectively prespecified. The underlying data and scoring documentation are provided in the supplementary file (S1 Data).

## Results

Table 1 shows that most participants were aged 18–22 years (57.9%), female (69.6%), and unmarried (90.6%). Most participants lived with their families (83.6%), reported sufficient monthly family income (77.9%), and had a normal BMI (53.0%). Health sciences students represented approximately half of the sample (50.4%). More than half of participants did not engage in physical activity (55.6%), were non-smokers (92.5%), and did not report a medically diagnosed chronic disease (92.7%). Regarding social media exposure, 57.1% reported spending more than four hours per day on social media.

**Table 1:** Sociodemographic, health, lifestyle, and social media use characteristics of participants (n = 385).

Variable	Category	n	%
Age group	18–22 years	223	57.9
	23–25 years	111	28.8
	>25 years	51	13.2
Gender	Female	268	69.6
	Male	117	30.4
Marital status	Unmarried	349	90.6
	Married	36	9.4
Residence	With family	322	83.6
	Alone	37	9.6
	With friends	26	6.8
Monthly Family Income	Sufficient	300	77.9
	Insufficient	85	22.1
BMI category	Underweight (<18.5 kg/m²)	59	15.3
	Normal weight (18.5–24.9 kg/m²)	204	53.0
	Overweight (25.0–29.9 kg/m²)	101	26.2
	Obesity (≥30.0 kg/m²)	21	5.5
Type of college	Health colleges	194	50.4
	Science and engineering colleges	76	19.7
	Humanities and social sciences Colleges	71	18.4
	Community and applied colleges	44	11.4
Academic level	First year	44	11.4
	Second year	58	15.1
	Third year	84	21.8
	Fourth year	119	30.9
	Internship Year	80	20.8
Chronic Illness	No	357	92.7
	Yes	28	7.3
Physical activity	No	214	55.6
	Yes	171	44.4
Smoking status	Non-smoker	356	92.5
	Smoker	29	7.5
Sleep duration	≤6 hours	133	34.5
	7-8 hours	184	47.8
	>8 hours	68	17.7
Following a Specific Healthy Diet	No	293	76.1
	Yes	92	23.9
Daily Social Media Use	<1 hour	9	2.3
	1-2 hours	39	10.1
	3-4 hours	117	30.4
	>4 hours	220	57.1

BMI: body mass index.

As shown in Table 2, the mean Dietary Habits score was 12.06 ± 4.17 (95% CI: 11.65–12.48) out of a possible score range of 5–25. Among the five-point IBM constructs, Perceived Norms (15.13 ± 6.07) and Intention (15.03 ± 5.75) demonstrated the highest mean scores, whereas Salience had a mean score of 11.58 ± 4.51 out of a possible score of 20. Skills and Knowledge, which were assessed using dichotomous items (score range: 0–4), had mean scores of 1.11 ± 1.43 and 2.22 ± 1.51, respectively. Internal consistency reliability was acceptable to excellent for all questionnaire constructs, with Cronbach’s alpha coefficients ranging from 0.783 for Knowledge to 0.931 for Intention.

**Table 2:** Descriptive statistics and internal reliability of the integrated behavioral model (IBM) constructs related to social media and dietary habits among the participants (n = 385).

IBM construct	Mean ± SD	95% CI of mean	Median	Min	Max	α
Dietary habits	12.06 ± 4.17	11.65-12.48	12	5	25	0.815
Attitude	13.87 ± 5.63	13.30-14.43	13	5	25	0.924
Perceived norms	15.13 ± 6.07	14.52-15.74	15	6	30	0.903
Personal agency	13.21 ± 5.32	12.68-13.74	12	5	25	0.915
Intention	15.03 ± 5.75	14.46-15.61	15	5	25	0.931
Salience	11.58 ± 4.51	11.13-12.04	12	4	20	0.876
Skills	1.11 ± 1.43	0.96-1.25	0	0	4	0.817
Knowledge	2.22 ± 1.51	2.07-2.37	2	0	4	0.783

SD: standard deviation; Min: minimum; Max: maximum; α: Cronbach’s alpha.

Table 3 demonstrates that Dietary Habits had statistically significant positive correlations with all seven other IBM constructs (all p < 0.001). Dietary habits showed the strongest correlations with attitude (rho = 0.62), perceived norms (rho = 0.61), personal agency (rho = 0.59), and skills (rho = 0.54), while the weakest correlation was observed with knowledge (rho = 0.31).

**Table 3:** Spearman correlation matrix of IBM constructs related to social media and dietary habits among the participants (n = 385).

Domain	Dietary Habits	Attitude	Perceived Norms	Personal Agency	Intention	Salience	Skills	Knowledge
Dietary habits	1.00	0.62**	0.61**	0.59**	0.48**	0.50**	0.54**	0.31**
Attitude	0.62**	1.00	0.84**	0.85**	0.74**	0.79**	0.70**	0.55**
Perceived norms	0.61**	0.84**	1.00	0.83**	0.68**	0.72**	0.65**	0.43**
Personal agency	0.59**	0.85**	0.83**	1.00	0.72**	0.79**	0.67**	0.50**
Intention	0.48**	0.74**	0.68**	0.72**	1.00	0.84**	0.62**	0.54**
Salience	0.50**	0.79**	0.72**	0.79**	0.84**	1.00	0.65**	0.55**
Skills	0.54**	0.70**	0.65**	0.67**	0.62**	0.65**	1.00	0.59**
Knowledge	0.31**	0.55**	0.43**	0.50**	0.54**	0.55**	0.59**	1.00

ρ: Spearman’s rank correlation coefficient; **p < 0.001.

Table 4 presents exploratory comparisons of the five-item Dietary Habits score, with significant differences according to gender, marital status, BMI category, chronic illness, physical activity, smoking status, sleep duration, following a healthy diet, and daily social media use (all nominal p < 0.05), whereas no significant differences were observed across age groups (p = 0.141). Female participants had significantly higher scores than males (12.60 ± 4.06 vs. 10.83 ± 4.15; p < 0.001), with a small-to-moderate effect size (Cohen’s d = 0.434). Married participants also reported higher scores than unmarried participants (13.64 ± 3.64 vs. 11.90 ± 4.19; p = 0.010). Dietary Habits scores varied significantly by BMI category (p < 0.001; η² = 0.105). Tukey’s HSD post hoc comparisons showed that normal-weight participants had significantly higher scores than underweight participants (mean difference [MD] = 1.90, 95% CI: 0.39–3.41, adjusted p = 0.007), overweight participants (MD = 2.67, 95% CI: 1.42–3.91, adjusted p < 0.001), and participants with obesity (MD = 3.94, 95% CI: 1.60–6.28, adjusted p < 0.001). Participants with chronic illness had a significantly higher Dietary Habits score than those without chronic illness (13.86 ± 3.76 vs. 11.92 ± 4.17; p = 0.014). Similarly, physically active participants had substantially higher scores than physically inactive participants (14.46 ± 3.84 vs. 10.15 ± 3.35; p < 0.001), with a large effect size (Cohen’s d = 1.206). Non-smokers also had significantly higher scores than smokers (12.19 ± 4.17 vs. 10.55 ± 3.87; p = 0.037). Dietary Habits scores also differed significantly by sleep duration (p = 0.011; η² = 0.023). Tukey’s HSD analysis indicated that participants who slept 7–8 hours per night had significantly higher scores than those who slept 6 hours or less (MD = 1.42, 95% CI: 0.31–2.52, adjusted p = 0.008), while no other pairwise differences were statistically significant. Following a healthy diet was strongly associated with higher Dietary Habits scores (15.60 ± 3.47 vs. 10.96 ± 3.73; p < 0.001), with a large effect size (Cohen’s d = 1.265). Finally, Dietary Habits scores differed significantly according to daily social media use (p < 0.001; η² = 0.131). Tukey’s HSD post hoc comparisons showed that participants using social media for 1–2 hours per day had significantly higher scores than those using it for more than 4 hours per day (MD = 4.06, 95% CI: 2.31–5.81, adjusted p < 0.001). Participants using social media for 3–4 hours per day also had significantly higher scores than those using it for more than 4 hours per day (MD = 2.55, 95% CI: 1.39–3.70, adjusted p < 0.001).

**Table 4:** Association of social media–related dietary habits scores with Participants’ sociodemographic, health, lifestyle, and social media use characteristics (n = 385).

Variable	Category	Dietary Habits Score Mean ± SD	Statistical test	p-value	Effect size	Tukey’s HSD Comparisons for Significant ANOVA Results
Age group	18–22 years	11.92 ± 4.46	F = 1.97	0.141	η² = 0.010	–
	23–25 years	11.86 ± 3.85				
	>25 years	13.14 ± 3.30				
Gender	Female	12.60 ± 4.06	t = 3.88	<0.001**	d = 0.434	–
	Male	10.83 ± 4.15				
Marital status	Unmarried	11.90 ± 4.19	t = −2.68	0.010*	d = 0.419	–
	Married	13.64 ± 3.64				
Monthly Income	Sufficient	12.15 ± 4.14	t = 0.77	0.444	d = 0.096	–
	Insufficient	11.75 ± 4.28				
Body Mass Index	Underweight (<18.5 kg/m²)	11.37 ± 4.16	F = 14.83	<0.001**	η² = 0.105	Normal weight vs underweight: MD = 1.90, 95% CI: 0.39–3.41, adjusted p = 0.007
	Normal weight (18.5–24.9 kg/m²)	13.27 ± 4.04				Normal weight vs overweight: MD = 2.67, 95% CI: 1.42–3.91, adjusted p < 0.001
	Overweight (25.0–29.9 kg/m²)	10.60 ± 3.74				Normal weight vs obesity: MD = 3.94, 95% CI: 1.60–6.28, adjusted p < 0.001
	Obesity (≥30.0 kg/m²)	9.33 ± 3.57				
Chronic Illness	No	11.92 ± 4.17	t = −2.60	0.014*	d = 0.467	–
	Yes	13.86 ± 3.76				
Physical Activity	No	10.15 ± 3.35	t = −11.58	<0.001**	d = 1.206	–
	Yes	14.46 ± 3.84				
Smoking Status	Non-smoker	12.19 ± 4.17	t = 2.18	0.037*	d = 0.394	–
	Smoker	10.55 ± 3.87				
Sleep Duration	≤6 hours	11.23 ± 4.21	F = 4.56	0.011*	η² = 0.023	7–8 hours vs **≤** 6 hours: MD = 1.42, 95% CI: 0.31–2.52, adjusted p = 0.008
	7–8 hours	12.64 ± 3.93				
	>8 hours	12.15 ± 4.48				
Following a Specific Healthy Diet	No	10.96 ± 3.73	t = −10.99	<0.001**	d = 1.265	–
	Yes	15.60 ± 3.47				
Daily Social Media Use	<1 hour	13.67 ± 3.74	F = 19.09	<0.001**	η² = 0.131	1–2 hours vs > 4 hours: MD = 4.06, 95% CI: 2.31–5.81, adjusted p < 0.001
	1–2 hours	14.87 ± 4.01				3–4 hours vs > 4 hours: MD = 2.55, 95% CI: 1.39–3.70, adjusted p < 0.001
	3–4 hours	13.36 ± 3.61				
	>4 hours	10.81 ± 4.03				

SD: standard deviation; t: Welch’s independent-samples t-test; F: One-way ANOVA; d: Cohen’s d; η²: eta squared; MD: mean difference; CI: confidence interval; *p < 0.05; **p < 0.01; No other pairwise comparisons were statistically significant. These group comparisons are exploratory; Tukey-adjusted pairwise p-values apply within each factor and do not control the overall family of group-comparison tests.

Table 5 illustrates that the primary model was statistically significant (F[32,352] = 14.92, p < 0.001) and explained 57.6% of the variance in dietary habits scores (adjusted R² = 0.537). After Holm adjustment across 32 coefficient tests, Attitude (B = 0.199, 95% CI 0.079–0.319, adjusted p = 0.034), physical activity (B = 1.295, 95% CI 0.536–2.054, adjusted p = 0.026), and social media use of 1–2 hours/day (B = 2.005, 95% CI 0.921–3.089, adjusted p = 0.010) and 3–4 hours/day (B = 1.220, 95% CI 0.511–1.929, adjusted p = 0.024), compared with >4 hours/day, were positively associated with Dietary Habits. No other coefficients remained statistically significant after Holm adjustment. VIF values ranged from 1.118 to 5.639, and tolerance values ranged from 0.177 to 0.894. Attitude exceeded the specified caution thresholds (VIF = 5.639; tolerance = 0.177), while Salience approached them (VIF = 4.951; tolerance = 0.202). All other predictors had VIF values below 5 and tolerance values above 0.20.

**Table 5:** Multiple linear regression analysis of factors associated with higher dietary habits scores (N = 385).

Predictor/comparison	B	SE	β	95% CI for B	t	Nominal p	Holm-adjusted p	Tolerance	VIF
Age 23–25 vs 18–22 years	0.496	0.401	0.054	−0.292 to 1.284	1.237	0.217	1.000	0.633	1.579
Age > 25 vs 18–22 years	0.306	0.572	0.025	−0.819 to 1.430	0.534	0.593	1.000	0.555	1.801
Male vs female	−0.356	0.374	−0.039	−1.091 to 0.378	−0.954	0.341	1.000	0.707	1.415
Married vs unmarried	0.550	0.541	0.039	−0.513 to 1.613	1.018	0.309	1.000	0.842	1.187
Underweight vs normal BMI	−0.247	0.464	−0.021	−1.160 to 0.667	−0.531	0.596	1.000	0.746	1.341
Overweight vs normal BMI	−0.483	0.393	−0.051	−1.256 to 0.290	−1.229	0.220	1.000	0.698	1.432
Obesity vs normal BMI	−2.166	0.700	−0.118	−3.542 to −0.790	−3.096	0.002	0.059	0.827	1.210
Chronic disease: yes vs no	0.150	0.605	0.009	−1.040 to 1.340	0.248	0.805	1.000	0.845	1.183
Physical activity: yes vs no	1.295	0.386	0.155	0.536 to 2.054	3.357	<0.001	0.026*	0.568	1.761
Smoker vs non-smoker	0.038	0.616	0.002	−1.173 to 1.250	0.062	0.951	1.000	0.790	1.267
Social media <1 vs > 4 hours/day	1.551	1.011	0.056	−0.438 to 3.539	1.534	0.126	1.000	0.894	1.118
Social media 1–2 vs > 4 hours/day	2.005	0.551	0.145	0.921 to 3.089	3.636	<0.001	0.010*	0.754	1.326
Social media 3–4 vs > 4 hours/day	1.220	0.360	0.135	0.511 to 1.929	3.386	<0.001	0.024*	0.760	1.316
Sleep 7–8 vs ≤ 6 hours/night	0.580	0.344	0.070	−0.096 to 1.256	1.687	0.092	1.000	0.708	1.412
Sleep >8 vs ≤ 6 hours/night	0.254	0.450	0.023	−0.631 to 1.139	0.565	0.573	1.000	0.708	1.412
Attitude	0.199	0.061	0.270	0.079 to 0.319	3.269	0.001	0.034*	0.177	5.639
Perceived Norms	0.132	0.051	0.192	0.031 to 0.232	2.578	0.010	0.258	0.218	4.593
Personal Agency	0.066	0.059	0.084	−0.051 to 0.182	1.109	0.268	1.000	0.211	4.747
Intention	0.045	0.051	0.062	−0.055 to 0.144	0.879	0.380	1.000	0.246	4.073
Salience	−0.126	0.071	−0.137	−0.267 to 0.014	−1.773	0.077	1.000	0.202	4.951
Skills	0.500	0.163	0.172	0.180 to 0.821	3.073	0.002	0.062	0.386	2.591
Knowledge	−0.293	0.131	−0.106	−0.550 to −0.036	−2.242	0.026	0.589	0.538	1.858
Living with friends vs family	0.938	0.657	0.057	−0.355 to 2.230	1.427	0.155	1.000	0.767	1.304
Living alone vs with family	1.502	0.538	0.106	0.444 to 2.561	2.791	0.006	0.144	0.829	1.206
Insufficient vs sufficient family income	−0.521	0.397	−0.052	−1.301 to 0.259	−1.314	0.190	1.000	0.771	1.297
Community/applied colleges vs health colleges	−0.422	0.525	−0.032	−1.455 to 0.610	−0.804	0.422	1.000	0.748	1.337
Humanities colleges vs health colleges	0.232	0.442	0.022	−0.637 to 1.101	0.524	0.601	1.000	0.711	1.407
Science colleges vs health colleges	0.783	0.430	0.075	−0.064 to 1.629	1.818	0.070	1.000	0.711	1.406
Internship vs fourth academic year	−0.264	0.456	−0.026	−1.161 to 0.634	−0.577	0.564	1.000	0.608	1.644
Third vs fourth academic year	−1.070	0.458	−0.106	−1.970 to −0.170	−2.338	0.020	0.478	0.584	1.712
Second vs fourth academic year	0.793	0.525	0.068	−0.239 to 1.825	1.512	0.132	1.000	0.592	1.688
First vs fourth academic year	−0.206	0.569	−0.016	−1.325 to 0.914	−0.361	0.718	1.000	0.636	1.572

Model fit: R² = 0.576; adjusted R² = 0.537; F(32, 352) = 14.92, p < 0.001; B, unstandardized coefficient; SE, standard error; β, standardized coefficient; CI, confidence interval; BMI, body mass index; VIF, variance inflation factor. All predictors were entered simultaneously using ordinary least squares. Following a healthy diet was excluded because of conceptual overlap with the outcome. Holm–Bonferroni adjustment was applied across the 32 slope-coefficient tests; 95% CIs are unadjusted for multiplicity. *Holm-adjusted p < 0.05 indicates statistical significance. Bold diagnostic values indicate VIF ≥ 5 or tolerance ≤0.20; these thresholds warrant cautious interpretation and do not establish coefficient stability.

## Discussion

Understanding the behavioral factors associated with social media–related dietary behaviors is important for informing future digital health promotion and digital nutrition communication strategies delivered through social media. Guided by the IBM, which explains health behaviors through the interaction of attitudes, perceived norms, personal agency, intentions, knowledge, and behavioral skills, the present study aimed to examine the relationship between social media use and dietary habits among university students. Specifically, the study assessed IBM constructs related to social media and dietary behaviors, explored their associations with dietary habits, and identified the sociodemographic, health, lifestyle, social media-related, and behavioral factors associated with dietary habits.

The descriptive findings showed variation across the IBM constructs, suggesting that social media–related dietary behaviors were associated with multiple IBM constructs. This pattern is consistent with the theoretical premise of the IBM, which emphasizes the combined roles of social influence, motivation, personal agency, knowledge, and behavioral skills in shaping health-related behaviors [18]. Through social media platforms, university students are regularly exposed to health information, including nutrition messages, peer recommendations, and other health-related content. Such digital communication may be relevant to attitudes, perceived norms, and motivation related to dietary behaviors [1]. Domain totals cannot be ranked as stronger or weaker performance because item counts and response formats differ. Denniss et al. reported frequent inaccuracies in nutrition-related Instagram information, supporting caution about equating online exposure with usable nutrition knowledge [32].

Previous reviews have examined social media as part of structured nutrition interventions, although their findings do not establish that passive exposure improves dietary behavior [1,33]. The strong positive connections among all parts of the IBM are consistent with its multidimensional structure. This theory suggests that health behaviors are shaped by several connected factors, such as thoughts, motivations, social influences, and the environment, rather than being caused by a single factor [18]. The observed pattern suggests that students with better nutrition-related abilities, more perceived social support, more personal agency, and more positive attitudes toward healthy eating tend to exhibit higher social media–related Dietary Habits scores. These results align with the IBM, which highlights the theoretically related roles of attitudes, perceived norms, self-efficacy, and knowledge in understanding behavioral intentions and actual behavior [18]. The weaker link between knowledge and dietary habits suggests that knowledge alone may not be sufficient to support healthy dietary behaviors. Additional motivation and personal control may also be relevant, as discussed in previous studies on nutrition behavior [19,20]. The positive correlations may reflect shared behavioral beliefs and common self-report measurement; they do not establish distinct constructs or causal pathways.

The exploratory comparisons indicate that social media–related Dietary Habits scores among university students differed across sociodemographic, health, and lifestyle characteristics. Higher scores among female students may reflect differences in health engagement or reporting, although these explanations were not assessed. The female-only studies by Malloy et al. and Sahin and Sanlier cannot establish a female–male difference [34,35]. Alrubaiee et al. reported more overweight or obesity among female Saudi students, illustrating that gender differences depend on the outcome assessed [6]. The lower Dietary Habits scores observed among participants with obesity are consistent with previous research reporting associations between excess body weight and less favorable dietary patterns, although the cross-sectional design prevents determination of directionality [36]. BMI-related differences may reflect eating patterns, weight-management efforts, or reverse directionality; the small obesity subgroup also limits precision. These exploratory differences should not be interpreted as independent associations, because gender and BMI were not statistically significant after covariate and Holm adjustment.

The finding that participants with chronic illness reported higher social media–related Dietary Habits scores may reflect greater motivation to adopt healthier lifestyles following medical advice or disease management; greater attention to dietary advice remains a possible explanation. Higher scores among married students may reflect more regular shared meals or household support; these factors were not measured. Non-smokers may share other health-conscious routines, while the sleep-duration difference may reflect daily routines and meal timing. Alrubaiee et al. linked smoking and sleep-related habits with weight status, offering contextual rather than direct support [6]. Following a healthy diet showed an exploratory association with Dietary Habits but was excluded from regression because of conceptual overlap. These group comparisons were exploratory, and Tukey’s HSD controlled pairwise comparisons within each factor rather than multiplicity across all group-comparison tests. Together, these results are compatible with the IBM, which provides a framework for examining personal, environmental, and behavioral correlates. They suggest that nutrition initiatives could consider education, everyday health practices, and the appropriate use of social media [18,33].

The multivariable regression analysis indicated that dietary habits were associated with both behavioral determinants and lifestyle characteristics. Positive attitude was the only IBM construct independently associated with higher social media–related Dietary Habits scores after covariate and Holm adjustment. Students who evaluate dietary advice positively may perceive it as more useful and compatible with their daily routines, making engagement with such information more personally meaningful. Zhang’s study likewise examined psychological correlates of college students’ dietary choices, illustrating the relevance of behavioral evaluations within a broader theoretical model [37]. Hammer et al.’s help-seeking-specific IBM adaptation also considers contextual conditions alongside attitudes, norms, and agency [38]. This theoretical perspective highlights the value of considering contextual conditions alongside individual behavioral beliefs. McDermott et al.’s meta-analysis identified attitudes as the strongest correlate of dietary intentions among the TPB constructs, providing a related theoretical comparison, although dietary intentions differ from the behaviors assessed here [20]. Douglas et al. reported an indirect association involving attitudes, perceived behavioral control, and diet quality among university students, suggesting that favorable evaluations may be considered alongside perceived control when interpreting dietary engagement [39]. Their dietary-quality outcome differs from the present social media–related score. The association may also reflect reciprocal relationships between favorable attitudes and previous dietary engagement; the cross-sectional design cannot establish directionality. The remaining IBM constructs were not independently significant after Holm adjustment, which does not establish that they are unimportant. Strong correlations among IBM constructs indicate substantial shared variance. Attitude’s elevated VIF suggests that multicollinearity may have reduced coefficient precision, and its Holm-adjusted association should therefore be interpreted cautiously. Salience also approached the specified caution thresholds. The coefficients represent associations conditional on the other included constructs, rather than each construct’s total association with Dietary Habits. These diagnostics do not demonstrate coefficient stability, and Holm adjustment addresses multiple testing rather than multicollinearity.

Physically active students had higher social media–related Dietary Habits scores than inactive students after covariate and Holm adjustment. This association may reflect the clustering of health-related practices, shared health motivation, or more structured daily routines. Shatwan and Alzharani similarly reported an association between higher physical activity and a higher Healthy Eating Index among Saudi college students, providing contextual support for the co-occurrence of physical activity and dietary practices [40]. However, their measure assessed dietary quality, whereas the present outcome assessed self-reported dietary behaviors related to social media. The observed association therefore does not establish that physical activity improves dietary behavior.

Students reporting 1–2 or 3–4 hours of daily social media use had higher Dietary Habits scores than those reporting >4 hours after covariate and Holm adjustment. This pattern may reflect differences in engagement, nutrition-related content, or daily routines. Bawazeer et al. also reported dietary-score differences according to social media duration among Riyadh students, although measurement and analytical approaches differed [41]. The smallest social media category may also have limited precision. Offering a contrasting perspective, Sahin and Sanlier associated social media addiction with healthy-eating obsessions and body-image concerns, highlighting that social media engagement may accompany problematic eating-related outcomes [35]. These outcomes differ from the present Dietary Habits score. The model’s explanatory fit does not demonstrate performance in other populations. Together, these findings support prospective evaluation of credible, theory-informed nutrition communication, rather than a prescribed duration of social media use. Malloy et al.’s co-design study informs acceptable communication approaches, while Klassen et al.’s review found inconsistent intervention effects, cautioning against assuming benefit [1,34]. Broader social media interventions have also been evaluated for health behavior change in low- and middle-income countries, providing implementation context rather than evidence for this specific dietary outcome [42]. The findings support considering the context of social media use when developing digital nutrition programs, without interpreting the observed duration categories as an optimal level of use.

### Strengths and limitations of the study

This study has several strengths. First, the IBM provided a theoretical framework for examining associations between social media–related dietary behaviors and behavioral constructs among university students. Second, the study included 385 students from multiple colleges and academic levels, providing variation in academic backgrounds within the study setting. Third, the use of a validated IBM-based questionnaire with domain-specific internal consistency provided evidence of score reliability. Finally, the findings provide theory-informed evidence that may support the development and evaluation of digital nutrition communication and health promotion strategies delivered through social media. Several limitations should be considered. The cross-sectional design cannot establish directionality or causation. Self-reported responses may be subject to recall and social desirability bias. The study was conducted at one university, limiting generalizability to other universities and cultural contexts. Convenience recruitment, particularly through social media, may have preferentially included students with greater platform engagement. This exposure-related selection bias may affect social media use distributions and observed associations. The predominance of women and health-college students further limits representativeness. Recruitment denominators and counts at earlier participation and exclusion stages were not recorded, preventing reconstruction of a complete recruitment flow and calculation of the overall response rate. The assessment of social media use focused primarily on duration rather than the quality, credibility, or types of nutrition-related content viewed. Residual confounding from unmeasured factors, such as nutrition literacy, psychological well-being, food availability, family eating practices, and socioeconomic environment, cannot be excluded. The Dietary Habits score assessed self-reported social media–related dietary behaviors and should not be interpreted as an objective or comprehensive measure of overall dietary quality. Following a healthy diet was excluded from the regression because of its conceptual overlap with the outcome. Strong intercorrelations among IBM constructs warrant caution regarding coefficient stability. Correlations and group comparisons were exploratory, and several nominally significant regression associations did not remain significant after Holm adjustment. The achieved-sample sensitivity assessment addressed overall-model power but did not establish adequate power or precision for individual regression coefficients, particularly for uncommon categories or correlated IBM constructs. Future longitudinal and multicenter studies incorporating objective dietary assessments and detailed measures of social media content are warranted to examine temporal relationships and improve generalizability.

## Conclusion

The study highlights the value of viewing social media–related dietary behaviors within students’ broader behavioral and lifestyle context. University nutrition communication could therefore consider how dietary messages relate to students’ attitudes and everyday health practices, alongside the nature of their engagement with social media. This perspective supports moving beyond exposure time alone when developing and evaluating digital nutrition initiatives. Future longitudinal studies could clarify how these relationships evolve over time and inform the development of nutrition initiatives suited to university students.

## Supporting information

S1 Data Supporting data for this study. It contains de-identified participant-level data, variable definitions and coding, domain-scoring procedures, and the regression model specification.
(XLSX)
